# International Regulations

**Published:** 1877-02-20

**Authors:** 


### INTERNATIONAL REGULATIONS.

It is said that the French government is about to prohibit American and other physicians foreign to their country, from practicing medicine in France. The same prohibition is being entertained in England, where an American physician was indicted for practicing without an English diploma. While it does not seem proper to permit, indiscriminately, as we do in the United States, every foreign pretender to practice the profession who may choose to do so, yet it would seem illiberal and unjust, if not inconsistent with international law, to ignore and disregard the rights which our diplomas confer upon us at home.

